# The mitochondrial genome of the endophyte *Edenia gomezpompae* CRI Eg3 isolated from sweet potato

**DOI:** 10.1080/23802359.2022.2048209

**Published:** 2022-03-06

**Authors:** Lifei Huang, Yaojia Mu, Xinxin Zhang, Kaijun Chang, Jiaming Zhang

**Affiliations:** a Crop Research Institute, Guangdong Academy of Agricultural Sciences, Guangdong Provincial Key Laboratory of Crops Genetics and Improvement, Guangzhou, Guangdong, China; b Hainan Key Laboratory of Microbiological Resources, Institute of Tropical Bioscience and Biotechnology, Hainan Bioenergy Center, Chinese Academy of Tropical Agricultural Sciences, Haikou, China

**Keywords:** *Edenia*, complete mitochondrial genome, endophyte, sweet potato

## Abstract

*Edenia gomezpompae* CRI Eg3 was isolated from the leaves of sweet potato. Its complete mitogenome contains 37,226 bp, with a G + C content of 26.1%. A total of 51 genes were annotated, including 16 protein-coding genes, 33 tRNA genes, and 2 rRNA (s-rRNA, L-rRNA) genes. The most significant character of this mitogenome is its free of group I introns in the CDS regions. Phylogenetic analysis using the mitogenomes of relative fungal species indicated that CRI Eg3 is closely related to *Shiraia bambusicola*, and they clustered in the Pleosporales lineage. This is the first genome reported in the genus Edenia.

*Edenia gomezpompae* Gonzalez et al. (2007) was described in 2007 (Gonzalez et al. 2007). The type strain was isolated from *Callicarpa acuminata* leaves, and was identified as an endophytic fungus. Similar strains were later isolated from leaves of *Senna alata* (Crous et al. 2009) and ginseng (Eo et al. 2014). The mycelium of *Edenia gomezpompae* produced naphthoquinone spiroketals with allelochemical, phytotoxic, antiparasitic and *in vitro* anticancer activities, and have potential application in fungicide (Macias-Rubalcava et al. 2008), herbicides (Macias-Rubalcava et al. 2014), and medicine (Martinez-Luis et al. 2011). *Edenia gomezpompae* CRI Eg3 was isolated from sweet potato leaves grown in Guangzhou city, Guangdong Province, China with geospatial coordinates N23°23′46″, E 113°26′36″. A specimen was deposited at the Guangdong Microbial Germplasm Preservation Center (http://www.gdmcc.net, Wang Yonghong, gdmcc@gdim.cn) under the voucher number GDMCC 3.705. The isolation process includes strict sterilization with 0.1% HgCl_2_ solution, followed by culturing on potato dextrose agar (PDA) medium. To investigate its taxonomic status and potential biological function, we sequenced its mitochondrial genome.

The genomic DNA was isolated as previously described (Ma et al. 2020, Yu et al. 2020), and sequenced using Illumina Hiseq 2500 and Nanopore platforms. The subreads were corrected with Canu v1.5 (Koren et al. 2017), assembled with wtdbg2 (Ruan and Li 2020), and further corrected using pilon (Walker et al. 2014). The final circular genome after removing the overlapped sequence was annotated with the MITOS webServer (http://mitos2.bioinf.uni-leipzig.de), and manually corrected using MacVector 13.6. The circular mitochondrial genome has a length of 37,226 bp, with a G + C content of 26.1%. A total of 51 genes were annotated, including 16 protein-coding genes, 33 tRNA genes, 2 rRNA (s-rRNA, L-rRNA) genes. The protein-coding genes include nine for NAD(P)H-quinone oxidoreductases (nad), one for ribosomal protein, one for ATP synthase, three for cytochrome oxidase subunits (coxs), one for cytochrome b (cob), and one for endonuclease. Transfer RNA genes for all 20 amino acids were identified. The most significant character in this mitogenome is that the protein coding sequences are all free of group I introns that are common in fungal mitochondrial genomes (Supplementary Figure S1).

Phylogenetic analysis using 12 peptides encoded by the mitogenomes of relative fungal species indicated that CRI Eg3 is most closely related to *Shiraia bambusicola*, and they clustered in the Pleosporales lineage (Figure 1).

**Figure 1.**
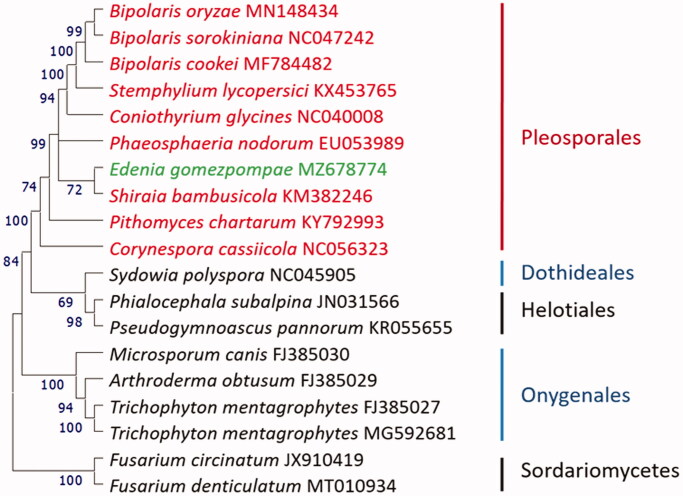
Maximum likelihood tree of *Edenia gomezpompae* and its relative fungal species. Coding DNA sequences shared by all taxa were extracted, translated, and combined in the same order before alignment with Clustal Omega (Sievers and Higgins 2018). 12 genes were used in the analysis (nad1, nad2, nad3, nad4, nad4 L, nad5, nad6, atp6, cob, cox1, cox2, cox3). Evolutionary history was inferred by using the maximum likelihood method and JTT matrix-based model (Jones et al. 1992), and rooted with two Hypocreales species in Sordariomycetes. Phylogenetic tree was prepared in MEGA11 (Tamura et al. 2016). Bootstrap supports for clades (1000 replicates) are shown above the branches.

## Supplementary Material

Supplemental Material

## Data Availability

The genome sequence data that support the findings of this study are openly available in GenBank of NCBI at https://www.ncbi.nlm.nih.gov under the accession no. MZ678774. The associated BioProject, SRA, and Bio-Sample numbers are PRJNA761904, SRX12120346, and SAMN21366478, respectively.”
