# Supplementary material for: The mitochondrial genome of the endophyte Edenia gomezpompae CRI Eg3 isolated from sweet potato
Source: Mitochondrial DNA B Resour. 2022 Mar 6;7(3):454–5. doi: 10.1080/23802359.2022.2048209 (PMC8903775; doi:10.1080/23802359.2022.2048209)
Supplement: Supplemental Material [file TMDN_A_2048209_SM9466.pdf]

Supplementary Figure 1. Alignments cytochrome oxidase subunit 1 gene of CRI Eg3 and closely related strain.

```
Cox1_Shiraia_bambusicola_KM382246     1  ATGTGATTTGAAAGATGATTTTTATCTTCAAATGCTAAAGATATTGGTGTATTATATTTAATATATGCTCTTTTTTCAGGGCTAGTTGGAACTGCGTTTT  100
Cox1_Edenia_gomezpompae_MZ678774      1  ATGTGATTTGAAAGATGATTTTTATCTTCAAATGCTAAAGATATTGGAGTATTATACTTAATATATGCTTTATTTTCAGGTTTAATAGGAACTGCCTTTT  100

Cox1_Shiraia_bambusicola_KM382246   101  CAGTATTGATAAGATTAGAATTGTCAGGGCCTGGAGTTCAGTACATTGCTGATAACCAATTATATAATAGTATTATAACAGCTCATGCTATTATTATGAT  200
Cox1_Edenia_gomezpompae_MZ678774    101  CTGTGTTAATAAGATTAGAGCTTTCAGGACCTGGTGTTCAGTATATTGCTGATAATCAATTATATAATAGTATTATTACTGCTCATGCTATTATTATGAT  200

Cox1_Shiraia_bambusicola_KM382246   201  TTTCTTTATGGTTATGCCAGCTTTAATAGGAGGTTTTGGAAATTTCCTACTTCCTTTAGGTTTAGGAGGTCCTGATATGGGATTCCCTAGACTTAATAAT  300
Cox1_Edenia_gomezpompae_MZ678774    201  TTTCTTCATGGTTATGCCAGCTTTAATTGGAGGATTTGGAAATTTTTTACTTCCTCTAGGATTGGGAGGTCCTGATATGGGATTCCCTAGACTTAATAAT  300

Cox1_Shiraia_bambusicola_KM382246   301  ATTAGTTATTTATTATTAATACCTAGTATTGTTCTTTTCTTATTTGCAGGTGGTATCGAAAACGGTGTAGGTACAGGATGAACTCTTTATCCACCGTTAT  400
Cox1_Edenia_gomezpompae_MZ678774    301  ATAAGTTACTTATTATTAATTCCTAGTATCGTTCTTTTCTTATTTGCAGGGGGGATAGAAAATGGTGTAGGTACAGGATGAACTCTATATCCACCTTTAT  400

Cox1_Shiraia_bambusicola_KM382246   401  CAGGTATACAAAGTCATAGTGGTCCAAGTGTTGATTTAGCAATATTTGGGTTACATCTTTCTGGTATATCTAGTCTTTTGGGGGCAATGAATTTTATGAC  500
Cox1_Edenia_gomezpompae_MZ678774    401  CAGGTATACAGAGTCATAGTGGTCCAAGTGTTGATTTAGCTATATTTGGGTTACATCTTTCTGGTATATCAAGTCTTCTTGGAGCCATGAATTTCATGAC  500

Cox1_Shiraia_bambusicola_KM382246   501  AACTACTTTTAATATGAGAAGTCCTGGAATAAGATTACATAAATTAATTTTATTTGCATGAGCAGTTGTTATTACAGCTGTTTTATTATTATTATCATTA  600
Cox1_Edenia_gomezpompae_MZ678774    501  AACTACATTTAATATGAGAAGTCCTGGAATAAGATTACATAAATTAATTTTATTTGCATGAGCGGTTGTTATTACAGCTGTTTTATTACTTTTATCTTTA  600

Cox1_Shiraia_bambusicola_KM382246   601  CCTGTTTTAGCAGGTGGAATTACTATGGTATTAACGGATAGAAACTTCAATACTTCATTCTTTGAAGTGGCTGGAGGGGGAGATCCAATTTTATACCAAC  700
Cox1_Edenia_gomezpompae_MZ678774    601  CCTGTTTTAGCCGGTGGAATTACAATGGTGTTAACAGATAGAAACTTTAATACTTCATTCTTTGAGGTAGCAGGTGGTGGTGATCCTATATTATACCAAC  700

Cox1_Shiraia_bambusicola_KM382246   701  ATCTTTTCTGATTCTTCGGTCACCCTGAGGTTATGTATGTAGGCCTCATAACGTTGCTATATGCTGGGACCCCTTTGCTCTGTATTTTCGAATACTCGAT  800
Cox1_Edenia_gomezpompae_MZ678774    701  ATCTTTTCTGATTCTTCGGTCATCCTGAGGTT--------------------------------------------------------------------  732

Cox1_Shiraia_bambusicola_KM382246   801  CTCATATGATGTAGTTAAAAATGTGGAAAAAAGAAGGATATCAGCAGGTAACCTTTTATGTATTAAAAGGGATACCTCAGAGACTTTACGCAACGAAATT  900
Cox1_Edenia_gomezpompae_MZ678774    733  ----------------------------------------------------------------------------------------------------  732

Cox1_Shiraia_bambusicola_KM382246   901  ATAGTAAATTCAGATAATTTAGAAAATGTTAAACATGTATCAAATATTGTCGCTGAAAATTTAGAAAATGTTAAGCATGAATCAAGTATTGTCGCTAAAA  1000
Cox1_Edenia_gomezpompae_MZ678774    733  ----------------------------------------------------------------------------------------------------  732

Cox1_Shiraia_bambusicola_KM382246  1001  ATTTAGAAAATGTTAAACGTATATCAGAGATTGTTCCTAAACATTTAAAACCTCTAAATAATGAAGAATTTTCTAATTATCTAGCAGGATGGATAGACGG  1100
Cox1_Edenia_gomezpompae_MZ678774    733  ----------------------------------------------------------------------------------------------------  732

Cox1_Shiraia_bambusicola_KM382246  1101  AAAAGGTCATTTTATTAATTATAATTTATTAATTATAGAATTTAATTATTTAGATTTATTTTTAGCCCAATATATAAGAAAAAAAATAGGTTATGGTAAA  1200
Cox1_Edenia_gomezpompae_MZ678774    733  ----------------------------------------------------------------------------------------------------  732

Cox1_Shiraia_bambusicola_KM382246  1201  ATAGTACTAGTTGCTGATAAAACAAGATATTTTTTAATGATAAATGGTAAAAAAGGTATATTACACGTTATTAACTTAGTAAATGGTAAATTAAAAACAA  1300
Cox1_Edenia_gomezpompae_MZ678774    733  ----------------------------------------------------------------------------------------------------  732

Cox1_Shiraia_bambusicola_KM382246  1301  ATTTGAGATTTGACCAAGCAGTTAATAAGTGATTAAATTATTATAAGTATTGAAATAAAGATCAAGATCCTAAATTTATTATAGACATAACCCGTAATTT  1400
Cox1_Edenia_gomezpompae_MZ678774    733  ----------------------------------------------------------------------------------------------------  732

Cox1_Shiraia_bambusicola_KM382246  1401  TGATAACAACCATTGATTGGCAGGGTTTTCTGATGCGCAGGGTAGTTTTCAATTAGAATTGAATGAACAGGTTTCATTATATGAGATAGAAATAAACTTA  1500
Cox1_Edenia_gomezpompae_MZ678774    733  ----------------------------------------------------------------------------------------------------  732

Cox1_Shiraia_bambusicola_KM382246  1501  AATTTTCAATTATACTACCCATATGATTATATATTGAAATCGGTACAAAAGTATTTAGGTGGAAACATTGAATTTAATAAAAGTCATTCAACTTATTATT  1600
Cox1_Edenia_gomezpompae_MZ678774    733  ----------------------------------------------------------------------------------------------------  732

Cox1_Shiraia_bambusicola_KM382246  1601  ATAACTCCATTACTTTTGGTTCTGCTAGAAATGTTATAAAATATTTTGATAAATACCACTTACAATCTACAAAATATTTAAGCTATTTAAAATGAAGACA  1700
Cox1_Edenia_gomezpompae_MZ678774    733  ----------------------------------------------------------------------------------------------------  732

Cox1_Shiraia_bambusicola_KM382246  1701  AGTATATAGCTTGTTACAAAACAAAAAAGATATAACAAAAAAAGAACTACTTAAAATACGTAGAATAGATTCTTCAATAAATAAATTCTAAATTCTTTAA  1800
Cox1_Edenia_gomezpompae_MZ678774    733  ----------------------------------------------------------------------------------------------------  732

Cox1_Shiraia_bambusicola_KM382246  1801  CAAATAGTTAATTTCTTAACAAATACTAAATTTTTTAACAAATATTATATTTTTTAACATATACTAAATTTTTTAACAAATACTGCTTTATAGTATGCTA  1900
Cox1_Edenia_gomezpompae_MZ678774    733  ----------------------------------------------------------------------------------------------------  732

Cox1_Shiraia_bambusicola_KM382246  1901  TATGTAAGATAAAGTCCCAACATAGATATAAGAAAAGTCTATGAGTATTAACAAAGACAGACTGTTAGAAAGCTGTCCTTATTAATCAAATGTTAGTTAC  2000
Cox1_Edenia_gomezpompae_MZ678774    733  -------------------------------------------------------------------------------------------------TAT  735

Cox1_Shiraia_bambusicola_KM382246  2001  ATTTTAATTATTCCTGGGTTTGGTATTATAAGTACAACTATCTCAGCTAACTCTAATAAAAGCGTTTTCGGTTACCTTGGTATGGTTTATGCTATGTGTT  2100
Cox1_Edenia_gomezpompae_MZ678774    736  ATTTTAATTATTCCAGGATTTGGTATAATAAGTACTACAATTTCAGCTAATTCTAACAAAAGTGTTTTTGGATACCTTGGAATGGTTTACGCCATGTGTT  835

Cox1_Shiraia_bambusicola_KM382246  2101  CTATTGGTATATTAGGATTTGTAGTTTGAAGTCATCATATGTACACTGTTGGTCTTGATGTAGATACAAGAGCGTATTTCACAGCTGCAACTTTAATTAT  2200
Cox1_Edenia_gomezpompae_MZ678774    836  CTATTGGTATTTTAGGGTTTGTAGTTTGAAGTCACCACATGTATACTGTTGGTCTTGATGTAGATACAAGAGCATATTTTACAGCTGCAACTTTAATTAT  935

Cox1_Shiraia_bambusicola_KM382246  2201  TGCAGTTCCTACAGGTATTAAAATCTTTTCTTGATTAGCAACTTGTTATGGTGGGTCTTTACACTTTATTCCATCATTATTATTTGCTCTAGGTTTCGTA  2300
Cox1_Edenia_gomezpompae_MZ678774    936  TGCAGTGCCTACTGGTATTAAAATATTCTCTTGATTAGCTACTTGTTATGGTGGTTCTTTACAATTTATACCATCATTATTATTTGCTTTAGGTTTTGTA  1035

Cox1_Shiraia_bambusicola_KM382246  2301  TTTATGTTTACTATTGGAGGGTTAAGTGGTGTAGTTCTTGCGAACGCATCACTTGATATTGCATTCCACGATACTTACTATGTTGTGGCTCATTTCCACT  2400
Cox1_Edenia_gomezpompae_MZ678774   1036  TTTATGTTCACAATTGGAGGGTTAAGTGGTGTAGTTCTTGCGAATGCATCACTTGATGTTGCTTTCCACGATACTTACTATGTAGTTGCTCACTTCCACT  1135

Cox1_Shiraia_bambusicola_KM382246  2401  ATGTTTTAAGTATGGGTGCTGTATTTGCCTTATTTAGTGGATGATATTTCTGAATACCTAAAATTTTAGGATTGGATTATAATCTACTATATTCAAAAGC  2500
Cox1_Edenia_gomezpompae_MZ678774   1136  ACGTTTTAAGTATGGGGGCTGTATTTGCCTTATATAGTGGATGATACTTCTGAATTCCTAAAATTTTAGGATTAGATTATAACTTAACTTACTCTAAAGC  1235

Cox1_Shiraia_bambusicola_KM382246  2501  TCATTTTTGAGTATTATTTACGGGGGTAAATTTAACATTCTTCCCTCAACACTTCCTTGGTTTACAAGGTATGCCACGTAGAATTAGCGATTACCCTGAT  2600
Cox1_Edenia_gomezpompae_MZ678774   1236  TCATTTCTGAGTTTTATTTATAGGGGTTAATTTAACTTTCTTCCCTCAACATTTCCTAGGATTACAAGGTATGCCTCGTAGAATTAGTGATTACCCTGAT  1335

Cox1_Shiraia_bambusicola_KM382246  2601  GCTTTTACAGGTTGAAACTTTATTAGTAGTATTGGTTCTATTATATCTGTAGCTGCAACAGCATTGTTCTTACACATTGTGTACTTACAACTTGTTAAAG  2700
Cox1_Edenia_gomezpompae_MZ678774   1336  GCTTTTACAGGTTGAAACTTTATTAGTAGTATTGGTTCTGTTGTATCTGTAATTGCCACAGCACTTTTCTTACATATTGTATATTTACAACTTGTAAAAG  1435

Cox1_Shiraia_bambusicola_KM382246  2701  GTAAAGCTATCTTTGGGTATCCATGAGCTGTTCCTCAATTATTTAGCGATTATCTACGTATACTTAAAGATAAATGTGCACCAGGATTAGAGTGAGCATT  2800
Cox1_Edenia_gomezpompae_MZ678774   1436  GTAAAGCTATCTTCGGATATCCTTGAGCCGTTCCTCAATTATTTAGTGATTACTTACGTATACTTAAAGATAAATGTGCTCCAGGTTTAGAATGAGCATT  1535

Cox1_Shiraia_bambusicola_KM382246  2801  ACATAACCCACCTAAACCTCACGCATTTACTAGCTTACCATTACAAAGTACTGGTATAGTAAGTTCTATTTTTTTAGGATTGTAA  2885
Cox1_Edenia_gomezpompae_MZ678774   1536  ACACAGCCCACCTAAACCTCACGCATTTACTAGTTTACCTTTACAAAGTAGCGGTGTATTAAGCTTTATTTTTATAGGA--ATAAG  1619
```
